# Complement Initiation Varies by Sex in Intestinal Ischemia Reperfusion Injury

**DOI:** 10.3389/fimmu.2021.649882

**Published:** 2021-04-01

**Authors:** Miaomiao Wu, Jennifer M. Rowe, Sherry D. Fleming

**Affiliations:** ^1^ Animal Nutritional Genome and Germplasm Innovation Research Center, College of Animal Science and Technology, Hunan Agricultural University, Changsha, China; ^2^ Division of Biology, Kansas State University, Manhattan, KS, United States

**Keywords:** LTB4, PGE2, mucosal injury score, MBL, properdin, sex, inflammation, C1q

## Abstract

Intestinal ischemia reperfusion (IR)-induced tissue injury represents an acute inflammatory response with significant morbidity and mortality. The mechanism of IR-induced injury is not fully elucidated, but recent studies suggest a critical role for complement activation and for differences between sexes. To test the hypothesis that complement initiation differs by sex in intestinal IR, we performed intestinal IR on male and female WT C57B6L/, C1q^-/-^, MBL^-/-^, or properdin (P)^-/-^ mice. Intestinal injury, C3b and C5a production and *ex vivo* secretions were analyzed. Initial studies demonstrated a difference in complement mRNA and protein in male and female WT mice. In response to IR, male C1q-, MBL- and P-deficient mice sustained less injury than male WT mice. In contrast, only female MBL^-/-^ mice sustained significantly less injury than female wildtype mice. Importantly, wildtype, C1q^-/-^ and P^-/-^ female mice sustained significant less injury than the corresponding male mice. In addition, both C1q and MBL expression and deposition increased in WT male mice, while only elevated MBL expression and deposition occurred in WT female mice. These data suggested that males use both C1q and MBL pathways, while females tend to depend on lectin pathway during intestinal IR. Females produced significantly less serum C5a in MBL^-/-^ and P^-/-^ mice. Our findings suggested that complement activation plays a critical role in intestinal IR in a sex-dependent manner.

## Introduction

Ischemia reperfusion (IR) stems from the interruption of organ blood flow and its subsequent restoration and contributes to pathology in a wide range of clinical conditions, ranging from hemorrhagic shock, stroke, myocardial infarction, and early organ transplant rejection (1–5). These diseases are major causes of disability and even mortality. While not as common, acute mesenteric ischemia represents a potentially fatal vascular problem with an overall mortality rate of 70% to 90% (6, 7). It is becoming increasingly appreciated that gender differences exist in several models of IR, such as kidney (8), myocardial (9), hemorrhage (10), and intestine (11). After cardiac or brain ischemic events, females sustain less tissue injury, but remain hospitalized longer with higher in-hospital mortality (12–15). Importantly, after heart attacks or strokes, more females are permanently disabled than males (16). Furthermore, whole genome RNA-seq analysis also showed sexual dimorphism in transcriptional response to myocardial ischemia (17).

Similar to clinical studies, female mice sustain reduced and delayed intestinal damage in multiple forms of IR and hemorrhage (18–20). Previous studies suggested that the damaging inflammatory response consists of both neutrophil and complement activation (21–23). Many studies demonstrated that arachidonic acid metabolism to eicosanoid production is critical for IR-induced intestinal damage and prostaglandins E_2_ (PGE_2_) was required for damage in male mice (22). However, a recent study demonstrated that intestines from female mice secrete potent chemo-attractants, the eicosanoid leukotriene B_4_ (LTB_4_) and C5a from the complement cascade (24). Though previous studies suggested a sexual dimorphism in eicosanoid production, the IR-induced difference between sexes in complement activation is unclear.

The complement system consists of over 30 soluble and membrane bound proteins that protect the host from invading pathogens (25). Three distinct primary pathways initiate complement activation: the antibody-antigen-binding activates complement through C1q; mannose binding protein (MBP) binding to carbohydrate triggers the lectin complement pathway; and the alternative pathway involves cleavage of Factor B (FB) and stabilization by the plasma glycoprotein, properdin (25). Complement components are not only produced in the hepatocytes and white blood cells, but also are produced by many other cell types, including intestinal epithelial cells. Importantly, mice express two forms of MBL, MBL-A and MBL-C and the MBL^-/-^ mice in the current study are deficient in both forms (26, 27). MBL-A is an acute phase protein highly expressed by the liver and the sera with maximal expression at ~30 hr post-lipopolysaccharide exposure (28, 29). Recent data suggests that the colon expresses MBL-A in response to chronic inflammation or infection (30). In contrast, the intestinal epithelial cells specifically express MBL-C (29, 31).

Recent studies demonstrated that the complement system has a central role in orchestrating inflammatory responses and IR-induced tissue injury (32) of multiple organs, including the intestine (7), kidneys (33), brain (34), and heart (35), of humans and animals (36). Previous studies characterized the reperfusion inflammatory response after intestinal ischemia by deposition of IgM and C3 in ischemic tissues (1, 37–39). Mice deficient in multiple complement components sustain reduced renal and/or cerebral IR induced injury to varying degrees (34, 40, 41). Importantly, IgM initiates intestinal, myocardial, and cerebral IR injury (38, 42, 43). Thus, initiated by antibodies, mesenteric IR damage was alleviated in C2-, C3-, C4-, and MBL-deficient mice (3, 7, 44).

The majority of studies described above used male mice. Using *ex vivo* assays, C3 activation was similar in C57B6L/and Balb/c male and female mice (45). However, the terminal pathway from C5b to C9 was not as robust in females compared with male mice indicating sex plays an important role in IR-induced complement activation. However, the differences *in vivo* remain unclear. Sexual dimorphism in the complement system poses interesting questions for immune biology response. Interestingly, accumulated research supports the idea that sex impact the immune system significantly (46) and complement activity may differ by gender (45). We hypothesized that the initiation of complement pathways may differ between males and females.

## Materials and Methods

### Mice

WT C57B/6 (# 000664), C1q deficient (C1q^-/-^; # 031675) and MBL-1/2-deficient (MBL^-/-^; #006122) mice were purchased from The Jackson Laboratory. The properdin deficient (P^-/-^) mice were provided by Dr. Wenchao Song, University of Pennsylvania with NIH grant support RO1 AI085596. All complement deficient mice were on the C57B6L/background. All mice were bred and maintained in the Division of Biology at Kansas State University, Manhattan, KS with a 12-h light/12-h dark cycle with a standard diet. Sex and age-matched (8 to 10 weeks old) mice were selected for experiments. All studies were approved by the Institutional Animal Care and Use Committee at Kansas State University and conducted according to the Animal Welfare Act and other federal statutes and regulations concerning animals and experiments involving animals.

### Treatment of Intestinal IR

The surgical protocol for intestinal IR was performed as previously described (22). Briefly, isoflurane (2-3% in oxygen) anesthetized mice were subjected to a midline laparotomy and administered buprenorphine (0.06 mg/kg) for pain relief per the institutional animal care and use committee requirement. After a 30 min equilibration period, a small, nontraumatic, vascular clamp (Roboz Surgical Instruments, cat. #RS-5420) was applied to the superior mesenteric artery and ischemia was visually confirmed by blanching of the mid-jejunum. After a 30 min ischemic phase, the clamp was removed and the intestine was allowed to reperfuse for 15, 30, 60, or 120 min in WT mice or 30, 60, or 120 min in mutant mice. Reperfusion was affirmed by observing the return of pinkish color to the intestine. Sham mice were subjected to the same surgical intervention but without occlusion. All procedures were performed with the mice breathing spontaneously and all mice were kept on a 37°C water-circulating heating pad to maintain body temperature. During the reperfusion period, mice were allowed to recover from anesthesia. Additional isoflurane was administered immediately prior to sacrifice. After sacrifice, serum and four sections of the mid-jejunum (2 cm each), beginning approximately 10 cm distal to the gastroduodenal junction, were harvested for histological evaluation, immunohistochemistry, RT-PCR, as well as eicosanoid and cytokine determination.

### Histology and Immunohistochemistry

After euthanasia, 2 cm mid-jejunum specimens were immediately fixed in 10% neutral buffered formalin phosphate, embedded in paraffin, cut transversely in 8 cm sections, transferred to positively charged slides, and stained with hematoxylin and eosin (H&E). The score of mucosal injury (SMI) for each slide was assessed using a six-tiered scale adapted from Chiu et al. (47), as described previously (48). Briefly, the average mucosal damage score of the mid-jejunum intestinal section was calculated by two well-trained observers unaware of the treatment. Each observer graded 90-150 villi on a scale of 0-6 with the following categories: Normal villi were assigned a score of zero; a score of 1 were assigned to villi with tip distortion; score 2 was assigned when Guggenheims’ spaces were present; a score of 3 was assigned to villi with slight disruption of the epithelial cells; villi in which the lamina propria was exposed but intact were scored as 4; villi with exuding lamina propria were scored as 5; and score 6 was assigned when the villi displayed hemorrhage or denuded. Photomicrographs were acquired from H&E-stained slides by using a 20X, 0.5 Plan Fluor objective on Nikon 80i microscope. Images were obtained by utilizing a Nikon DS-5 M camera with the DS-L2 software at room temperature (Nikon).

Immediately after removal, additional 2 cm mid-jejunum sections were snap-frozen in optimal cutting temperature (O.C.T.) freezing medium (Fisher cat. # 23730571). Specimen were cut transversely in 8 cm sections and placed on the slides for immunohistochemistry, as described previously (23). Briefly, pre-chilled acetone fixed slides were incubated with 10% normal donkey sera in phosphate buffered saline (PBS) for 30 min at 37°C to block nonspecific binding. Following PBS washes, the intestinal sections were incubated with primary antibodies in the dark for 1 h at room temperature (RT) or overnight (O/N) at 4°C. Deposition of C3b, C1q, MBL-C, Factor B, and IgM deposition on the tissue sections was determined by staining with a purified rat-anti-mouse anti-C3b antibody (Hycult Biotechnologies, cat. # HM1065), anti-C1q or anti-MBL-C antibody (Cedarlane, cat. # CL7501AP and #CL7303AP, respectively), or rabbit-anti-mouse anti-Factor B antibody (Abcam, ab231072) followed by an Alexa Flour 594 conjugated donkey-anti-rat IgG secondary antibody (Jackson ImmunoResearch, cat. # 712586153) or FITC-green conjugated donkey-anti-rabbit IgG secondary (Jackson ImmunoResearch, cat. # 711096152). Some tissues were stained with a primary rat-anti-mouse MBL-A (Invitrogen MA5-33352) followed by an Alexa Fluor 594 conjugated donkey-anti-rat IgG secondary antibody. The detection of IgM was performed similarly with the exception of using FITC AffiniPure Fragment goat-anti-mouse IgM antibody (Jackson ImmunoResearch, cat. # 115096075), and no secondary antibody was needed. All experiments contained serial sections stained with the appropriate isotype control antibodies. The slides were then mounted with ProLong Gold (Invitrogen, cat. # P10144). Images (4-8 images per section) were acquired by a well-trained blinded observer at RT by using a Nikon eclipse 80i microscope equipped with an Infinity 3S camera (Lumenera) and analyzed by utilizing Infinity Analysis software (Lumenera). All microphotographs were examined by Image J software (National Institutes of Health) using the fluorescent area fraction after setting threshold for each experiment. The average of the isotype control was subtracted from each image. The average of 5 images from 3-4 animals per treatment group is analyzed and reported.

### *Ex Vivo* Cytokine and Eicosanoid Determination

Mid-jejunal tissue *ex vivo* generation of eicosanoids and cytokines was determined by using an adapted procedure previously described (49). Briefly, immediately after collection, 2 cm mid-jejunum sections were minced, washed in pre-chilled Tyrode buffer (Sigma-Aldrich), and resuspended in 37°C freshly oxygenated Tyrode buffer. After 20 min incubation at 37°C, the supernatants and tissues were collected and stored at -80°C until assayed. The tissue protein content was detected by utilizing the bicinchoninic acid assay (Pierce, cat. # 23225) modified for use with microtiter plates. Bovine Serum Albumin (BSA) (Fisher Scientific, cat. # BP9703100) was used as a standard. The same intestinal tissue supernatants were used to determine concentrations of leukotriene B_4_ (LTB_4_) and prostaglandins E_2_ (PGE_2_) utilizing enzyme immunoassay kits (Cayman Chemical, cat. # 520111 and #500141, respectively). Supernatants generated for eicosanoids were also used to detect cytokine levels by using Milliplex MAP immunoassay kits (Millipore, custom kits) that were analyzed on a Milliplex Analyzer (Millipore). The concentrations of eicosanoid and cytokines were standardized per mg intestinal tissue protein per 20 min incubation period.

### Serum IgM and C5a Determined by ELISA

Blood was collected from untreated or intestinal IR treated mice for IgM and C5a determination, respectively. Blood was allowed to clot for 30 min at room temperature and transferred to ice prior to serum collected by centrifugation for 20 min at approximately 1000×g. The mouse IgM (cat. # E99-101) and C5a (cat. # DY2150) were purchased from Bethyl Laboratories and R&D SYSTEMs, respectively. These assays were performed in accordance with the manufacturer’s recommended procedures.

### Quantitative Real-Time PCR

After euthanasia, one 2 cm mid-jejunal tissue approximately 10 cm distal to gastroduodenal junction was immediately snap frozen in liquid nitrogen and stored at 80°C until used for RNA analysis. Total RNA was isolated from intestine tissues using TRIzol reagent (Invitrogen, cat. # 15596018) and then treated with DNase I (Invitrogen, cat. #18047019) according to manufacturer’s instructions. RNA quantity and quality were evaluated and measured by utilizing the Nano drop (Thermo Fisher Scientific). The reverse transcription was conducted at 25°C for 5 min, 42°C for 30 min, and 85°C for 5 min to get cDNA with 1 µg total RNA by using qScript first strand cDNA synthesis kit (Quanta Biosciences, cat. # 95047). Primers (**Table 1**) were designed with Primer3 according to the mouse gene sequence. Quantitative real-time PCR was conducted in a 25 µL total volume using an Applied Biosciences StepOnePlus thermocyclers (Thermo Fisher) with Perfecta SYBR Green Fastmix reagent (Quanta Biosciences, cat. # 95072-250). The PCR protocol is as follows: (i) pre-denaturation for 3 min at 95°C; (ii) an amplification and quantification program including 50 cycles of 10 sec at 95°C, 20 sec at Tm (**Table 1**), 10 sec at 72°C; (iii) melt curve start at 60°C, increasing by 0.5°C up to 95°C. After amplification, 18s rRNA was used as an internal control to normalize relative gene transcript levels, ΔΔCt fold change relative to Sham-treated wildtype mice was determined.

**Table 1:** Real Time PCR Primer Sequences.

Gene	Tm (°C)^a^	Sequence
*C1qa*	58	FWD: CAC CAA CCA GGA GAG TCC AT
		REV: ACC TGA AAG AGC CCC TTG TT
*MBL1*	60	FWD: AGGGTCACAAACCTGTGAGG
		REV: TTTGCCAGCTTCTCCTCAAT
*MBL2*	56	FWD: ATTGCCTACTTGGGCATCAC
		REV: ATCGTTCCACTTGCCATTTC
*Factor B*	58	FWD: CCA GCA TTT GGG TTT CAG TT
		REV: CAC ACC TCC AGA GGA GAA GC
Ribosomal 18s^b^	56	FWD: GGTTGATCCTGCCAGTAGC
		REV: GCGACCAAAGGAACCATAAC

a Annealing temperature.

b House-Keeping gene to which genes of interest were normalized.

### Statistical Analysis

Data are presented as mean ± the standard error of the mean (SEM), and were statistically analyzed by 2-way ANOVA, 1-way ANOVA with Neman Keuls *post-hoc* test to compare between strains of mice with in the same sex, and unpaired t-test to determine differences between male and female of the same treatment. (Graph Pad/Instant Software Inc.). Differences between groups were considered significant when *P* value *≤* 0.05.

## Results

### Complement Initiation in Response to Intestinal IR Differs Between Sexes

**Figure 1:**
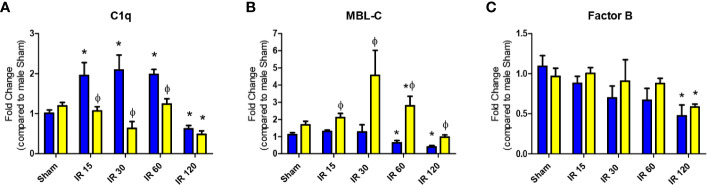
Intestinal IR-induced complement initiation transcription differs between sexes. C57B6L/J mice were subjected to Sham and IR treatment with 15, 30, 60, or 120 min reperfusion. Mid-jejunum of **(A)** C1q, **(B)** MBL-C, and **(C)** Factor B, RNA expression was determined by RT-PCR analysis. Samples were normalized to 18S followed by fold change compared to Sham treatment. *, indicates significant difference between IR treatment and Sham within the same sex (P ≤ 0.05), as determined by 1-way ANOVA followed by Newman Keuls *post-hoc* test; Ф, indicates significant difference between sexes in the same time point and same treatment (P ≤ 0.05), as determined by unpaired t-test. Each bar is representative of 7-12 mice per group.

Initiation of complement activation occurs by multiple pathways, which may lead to distinct complement activation in male vs female mice during intestinal IR. Initial studies examined complement initiation mRNA expression in intestines of wildtype male and female mice by RT-PCR of *C1qa, MBL-1*, *MBL-2*, and *Factor B.* The intestinal expression of C1q increased significantly in males at 15 min and remained elevated until 60 min IR (**Figure 1A**, blue bars), while female intestine expression of C1q remained similar to Sham levels for each of the time points examined (**Figure 1A**, yellow bars). C1q mRNA expression decreased at 120 min IR in both male and female intestines (**Figure 1A**). Intestinal expression of MBL-A mRNA was not different between sexes and in response to IR decreased at 60 min reperfusion (**Supplementary Figure 1**). In contrast, intestinal MBL-C expression did differ between sexes. In male intestines MBL-C was not elevated after IR, and significantly decreased from 60-120 min compared to intestines from Sham-treated males (**Figure 1B**, blue bars). However, the expression of intestinal MBL-C in females increased significantly from 30-60 min before decreasing significantly at 120 min IR (**Figure 1B**, yellow bars). Compared to males, female intestine express significantly higher level of MBL-C in Sham animals, and in IR from 30-120 min (**Figure 1B**). Both males and females had a significantly decreased level of intestinal FB mRNA at 120 min IR (**Figure 1C**). There were no significant differences in FB expression between male and female intestines, during the IR time course (**Figure 1C**).

**Figure 2:**
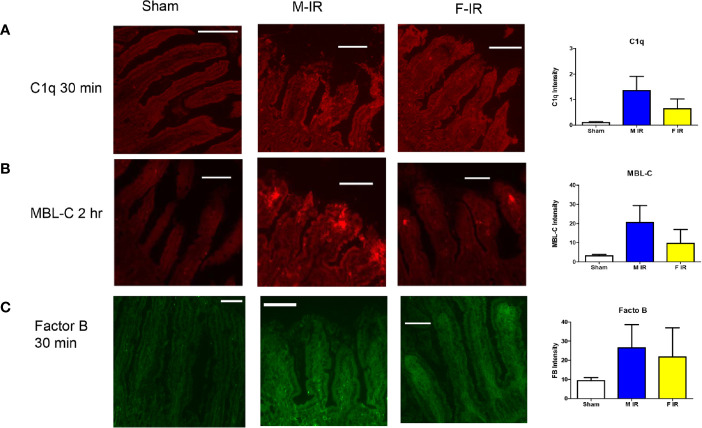
Intestine tissue C1q, MBL-C, Factor B, deposition differs between sexes. C57B6L/J mice were subjected to Sham and IR treatment with 30 or 120 min reperfusion. Intestinal sections were stained for **(A)** C1q, **(B)** MBL-C, **(C)** Factor B, by immunohistochemistry. Bar = 40 µm. The image analyses for each animal were determined by digital densitometry recognition using computer-aided ImageJ (NIH). Microphotographs (200X) are representative of at least 3-4 mice stained in at least 3 independent experiments.

To confirm the expression of complement initiators and major players in the complement pathways, we detected the intestinal deposition of C1q, MBL or MBL-C, and FB by immunohistochemistry. Similar to mRNA expression levels, C1q intestinal staining was slightly but not significantly increased in males at 30 min after ischemia and returned to Sham levels by 120 mins after ischemia (**Figure 2A** and data not shown). In contrast to C1q, minimal MBL-A or MBL-Cstaining was detected after 30 min of reperfusion (data not shown) but by 120 min, MBL-C accumulated on intestines of both sexes (**Figure 2B** and **Supplementary Figure 1**). These data correlate with previously reported intestinal expression of MBL-C but not MBL-A (29). Compared to the MBL-C staining, MBL-A staining was not increased compared to Sham treatment (**Supplementary Figure 1**). Although not significantly different, male intestines tended to have more - staining for MBL-C than females (**Figure 2B**). In addition, in both sexes antibodies directed against FB stained the intestines consistently after Sham or at any IR time points (**Figure 2C**).

**Figure 3:**
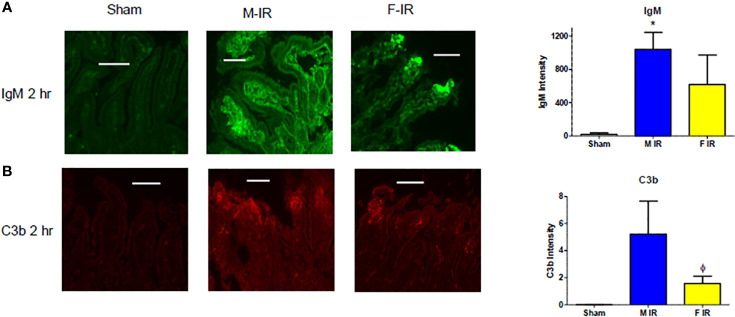
Intestine tissue IgM, and C3b deposition after 2 h reperfusion differs between sexes. C57B6L/J mice were subjected to Sham and IR treatment with 30- or 120-min reperfusion. Intestinal sections from Sham or IR WT mice were stained for **(A)** IgM and **(B)** C3b by immunohistochemistry. Bar = 40 μm. The image analyses for each animal were determined by digital densitometry recognition using computer-aided ImageJ (NIH). Microphotographs (200X) are representative of at least 3-4 mice stained in at least 3 independent experiments. *, indicates significant difference between IR treatment and Sham within the same sex (P ≤0.05), as determined by 1-way ANOVA followed by Newman Keuls *post-hoc* test; Ф, indicates significant difference between sexes in the same time point and same treatment (P ≤ 0.05), as determined by unpaired t-test.

In response to IR, IgM was deposited in both males and females after 120 min reperfusion (**Figure 3A**). Intestinal C3b deposition was enhanced in both sexes by 60 min (data now shown) and 120 min post-ischemia (**Figure 3B**). Taken together, our data indicate that all three complement pathways play a role in both sexes after intestinal IR in wildtype mice. However, during reperfusion, intestines in males contained increased C1q and MBL-C proteins, while females contained MBL-C. The amplification pathway as indicated by FB is important for IR-induced injury in both sexes of wildtype mice.

### Complement Deficiency Protects Mice From Intestinal IR Injury in a Sex Dependent Manner

**Figure 4:**
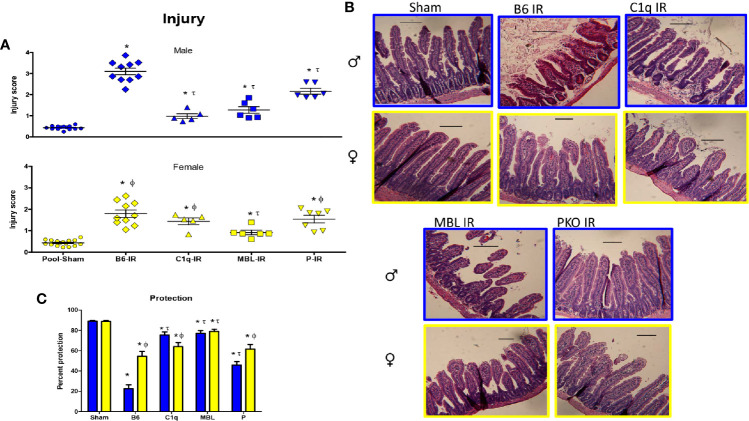
Intestinal IR-induced tissue damage was protected in complement deficient mice in a sex-dependent manner. C57B6L/J WT, C1q^-^/^-^, MBL^-^/^-^, and P^-^/^-^ mice were subjected to Sham and IR treatment with 120 min reperfusion and mid-jejunum sections H&E stained and scored for mucosal injury (75-150 villi per section). **(A)** Individual injury scores of Pooled Sham (Sham), or IR treated C1q^-/-^, MBL^-/-^ or P^-/-^ male (blue bars) and female (yellow bars) mice are shown. **(B)** Representative microphotographs of H&E stained section (200x) of Sham or IR treated C1q^-/-^, MBL^-/-^ or P^-/-^ male (blue outline) and female (yellow outline) mice are presented. Bar = 40 μm. **(C)** Percent protection was calculated by the following equation, (1-(Injury score/Max injury of 4.0)*100). * indicates significant difference between IR treatment and Sham within the same sex (P ≤0.05), as determined by 1-way ANOVA with Newman Keuls *post-hoc* test; Ф indicates significant difference between sexes in the same point same treatment (P ≤ 0.05), as determined by unpaired t-test; τ indicates significant difference between same sex IR compared to WT IR (P ≤ 0.05), as determined by 1-way ANOVA with Newman Keuls *post-hoc* test. Each bar is representative of 7-12 mice per group.

To understand the specific role of each initiation pathway intestinal IR-induced tissue damage, we subjected C1q**^-/-^**, MBL^-/-^, and P^-/-^ male and female mice to Sham or 120 min mesenteric IR. As there was no significance among Sham treatment at different time points of the same sex of either C57B6L/(wildtype, WT) or knockout (KO) mice, injury scores and subsequent analyses were pooled by sex for Sham treatment only (**Supplementary Table 1**). Compared to Sham mice, intestinal damage increased significantly in both sexes in WT mice after 120 min of reperfusion (**Figures 4A, B**). In addition, both sexes of C1q**^-/-^**, MBL**^-/-^**, and P**^-/-^** mice sustained increased tissue damage compared to the Pooled Sham animals. Compared to male WT after IR, intestinal injury was significantly reduced in all deficient mice (**Figure 4A**). In contrast, compared to the female WT after IR, intestinal injury in females was significantly decreased in only MBL**^-/-^**(**Figure 4A**). However,.a significant difference between sexes existed in all strains of animals. When comparing IR-induced damage in and males and females, WT and P^-/-^ male mice sustained significantly more tissue injury than female mice. However, deletion of C1q resulted in significantly reduced intestinal damage in males, compared to similarly treated female mice (**Figure 4A**).

Using the same data, the injury score was analyzed as a percent protection. Analysis by 2-way ANOVA indicated that the interaction of sex with the strain was extremely significant (**Figure 4C**). Although the complement deficiency accounted for the majority of the variance, sex was also significantly different. In response to IR, all strains of complement deficient male mice were more protected than the wildtype, C57B6L/mice (**Figure 4C**). However, wildtype female mice were protected ~55% compared to 25% protection in male C57B6L/mice. Although the complement deficient female mice were 60-80% protected, only the MBL-/- females were statistically significant compared to the wildtype (**Figure 4C**).

These data suggest that males are protected with any of the examined complement deficiencies and sustained an intermediate amount of injury in the absence of properdin. In contrast, only MBL^-/-^ female mice are significantly more protected than the wildtype mice. However, the female wildtype mice are more protected than the male wildtype mice. Together with changes in deposition of C1q, MBL, and FB (**Figure 2**), these results indicate that all three complement pathways are required for inducing intestinal IR injury in male mice, while the lectin pathway is particularly critical for inducing tissue damage in females. Specifically, IR-induced tissue damage requires C1q in males more than females, MBL is critical for both sexes, and alternative pathway appears to amplify the injury during intestinal IR.

### Depletion of MBL or Properdin Decreases Serum C5a Levels in Both Sexes Following Intestinal IR

**Figure 5:**
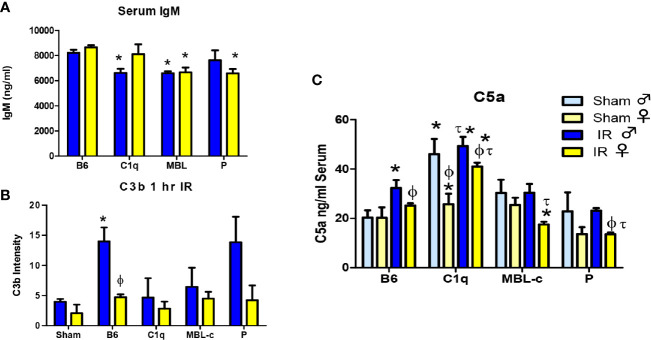
MBL-C or Factor P deficiency decreases serum IgM, C3b and C5a levels in both sexes following intestinal IR. **(A)** Serum from untreated C57B6L/J WT, C1q^-^/^-^, MBL^-^/^-^, and P^-^/^-^ male (blue) and female (yellow) mice was analyzed by ELISA for IgM. Sham treatment is indicated by light colors whereas IR is indicated by dark colors. Each bar represents 2-3 pools of 3-5 mice. **(B)** C3b deposition on intestines from male (blue) and female (yellow) C57B6L/J WT, C1q^-^/^-^, MBL^-^/^-^, and P^-^/^-^ mice was examined after Sham and IR treatment with 120 min reperfusion. Each bar represents 3-4 animals in at least 3 independent experiments **(C)** Serum C5a from C57B6L/J WT, C1q^-^/^-^, MBL^-^/^-^, and P^-^/^-^ mice was determined after Sham and IR treatment with 120 min reperfusion. Sham treatment represents pooled analysis of all strains. Each bar is representative of 7-12 mice per group. Serum IgM and C5a were measured with sandwich ELISA. C3b deposition was quantitated by Image J analysis after IHC. * indicates significant difference between IR treatment and Sham within the same sex (P ≤0.05), as determined by 1-way ANOVA with Newman Keuls *post-hoc* test; Ф indicates significant difference between sexes in the same time point same treatment (P ≤ 0.05), as determined by unpaired t-test; τ indicates significant difference between same sex IR compared to WT IR (P ≤ 0.05), as determined by 1-way ANOVA with Newman Keuls *post-hoc* test.

As IgM deposition initiates complement activation in response to IR, we examined normal serum IgM to determine if sufficient IgM was available for complement activation. Although all three genetically modified strains of mice contained significantly less serum IgM (**Figure 5A**), it is likely that with over 6000 ng/ml, sufficient IgM was available to activate complement. Complement activation leads to series of proteolytic amplifying events, including the cleavage of C3 and C5, which are common to all three-initiation pathways. Cleavage of each component results in deposition of C3b or C5b respectively on the tissue. As demonstrated in **Figure 3** and **Supplementary Figure 2**, wildtype, C57B6L/mice had significantly more C3b deposited on intestinal tissue in male mice than female mice. Importantly 2-way ANOVA indicated a significant difference based on sex but not on strain or the interaction of sex with the strains. Although not significant at 1 hr post ischemia, measurement of C3b deposition on intestinal tissue in complement deficient male mice was slightly decreased compared to staining of wildtype tissue. Thus, it appears that the multiple initiation pathways can compensate for the deletion of C1q, MBL or properdin in male mice. In contrast, the reduction of C3b in female mice after 1 hr reperfusion was not significantly different from Sham treated animals in either wildtype or complement deficient mice.

Similar to C3 cleavage, C5 cleavage generates an anaphylatoxin, C5a that is critical for initiation of neutrophil recruitment and activation and instigates increased IR-induced tissue damage (50–52). Thus, we examined C5a levels in the blood. C5a was the only analyte that differed significantly between sexes in Sham treated animals (**Supplementary Table 1**). Therefore, Sham treatments were not pooled for C5a. **Figure 5C** demonstrates that Sham-treated, male C1q^-/-^ mice produced significantly more C5a compared to corresponding male Sham-treated mice. In response to IR, serum C5a increased in both sexes of WT and female C1q**^-/-^** mice. In contrast, C5a concentrations in IR-treated MBL^-/-^ or P^-/-^ mice decreased or remained similar to Sham treatment (**Figure 5C**). Importantly, after IR, the serum C5a in females was consistently significantly lower when compared to males from the same strain. Although there was no significant difference in the interaction of sex with the strains, 2-way ANOVA indicated significant differences between both sexes and strains. Taken together, our data suggest that 1) generally, females express less C5a in response to Sham or IR treatment than males, 2) deletion of MBL or properdin decreases serum C5a levels in both sexes after IR and 3) deletion of C1q does not change serum C5a.

### Complement Deficiency Decreases Intestinal Inflammatory Secretions in a Sex Dependent Manner

**Figure 6:**
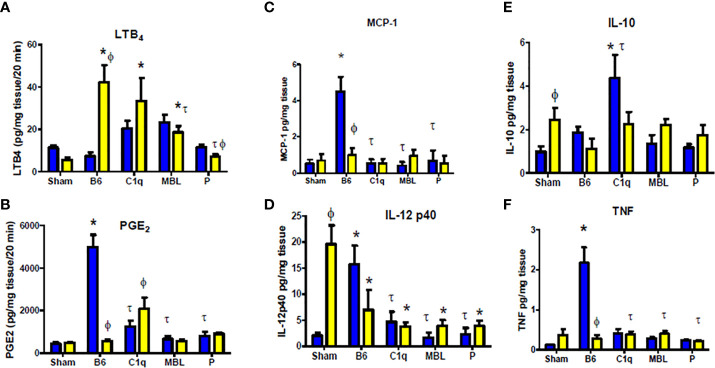
Complement deficiency decreases intestinal inflammatory secretions in a sex dependent manner. Male (blue) and female (yellow) C57B6L/J WT, C1q^-/-^, MBL^-^/^-^, and P^-^/^-^ mice were subjected to Sham (pooled from all strains) or IR treatment with 120 min reperfusion. Intestinal sections were analyzed *ex vivo* for **(A)** LTB_4_, **(B)** PGE_2_, **(C)** MCP-1, **(D)** TNF-α, **(E)** IL-12p40and **(F)** IL-10 production and standardized to mg total tissue protein. * indicates significant difference between IR treatment and Sham within the same sex (P ≤ 0.05), as determined by 1-way ANOVA followed by Newman Keuls *post-hoc* test; Ф indicates significant difference between sexes in the same time point same treatment (P ≤ 0.05), as determined by unpaired t-test; τ indicates significant difference between same sex IR compared to WT IR (P ≤ 0.05), as determined by1-way ANOVA with Newman Keuls *post-hoc* test. Each bar is representative of 3-7 mice per group.

Previous studies showed that eicosanoids, LTB_4_ and PGE_2_ are rapidly elevated in response to intestinal IR (53, 54). To confirm the role of C1q, MBL, and properdin in the inflammatory response to mesenteric IR, we examined *ex vivo* generation of both eicosanoids in intestine tissues after IR in all deficient mice. Similar to previous studies (24), intestines from IR treated WT female mice generated significantly elevated LTB_4_ at 120 min reperfusion, while LTB_4_ production was not increased in WT male mice when compared to male Sham treated mice (**Figure 6A**). Deletion of C1q or MBL, in male mice significantly increased the production of LTB_4_ to similar levels as found in female mice (**Figure 6A**). In contrast, IR-treated intestines from P^-/-^ male or female mice produced LTB_4_ similar to Sham treatment (**Figure 6A**). The interaction of sex with strains as well as both sex and strains differed significantly as determined by 2-way ANOVA.

Similar to our previous studies (24), intestines from IR treated WT male mice generated significantly elevated PGE_2_ at 120 min post-ischemia, while WT female mice maintained low PGE_2_ production similar to Sham-treated mice and significantly lower than male mice (**Figure 6B**). Deletion of C1q, MBL, or properdin in male mice significantly reduced production of PGE_2_ (**Figure 6B**). However, there was minimal change in the PGE_2_ production in female MBL^-/-^ or P^-/-^ mice compared to the corresponding WT mice (**Figure 6B**). Although increased in response to IR in C1q-/- females, PGE_2_ production remained less than half of the quantity produced by intestines from WT male mice (**Figure 6B**). Similar to LTB_4_, 2-way ANOVA indicated that the interaction of sex and strains differed significantly.

Intestinal ischemia/reperfusion induces TLR4 and subsequent pro-inflammatory cytokines that alter the inflammatory cellular response as well (22, 55). Intestines from WT male mice produced significantly increased MCP-1 (CCL2) and TNF-α which was not produced by intestines from WT females or any complement deficient mice (**Figures 6C, D**). In contrast, intestines from females of any strain of Sham treated mice produced significant quantities of IL-12p40 that was significantly decreased with IR. Importantly, IL-12p40 production by IR treated intestines from male WT mice was significantly increased compared to Sham treatment and was comparable to Sham-treated females. Mice of either sex with deficiency in complement components, C1q, MBL or P, reduced IL-12p40 production after IR. *Ex vivo* intestinal IL-10 production was also elevated in females after Sham treatment with no increased in response to IR in wildtype or complement deficiency. In contrast, male IL-10 expression increased in response to IR in WT and was significantly elevated in C1q^-/-^ mice but not in MBL^-/-^ or P^-/-^ mice. While the specific cytokine response differed by sex and by strain of mice, the interaction of sex with the mouse strain was significantly different in each cytokine. In addition, 2-way ANOVA indicated that sex was not significantly different in intestinal IL-12p40 and IL-10 secretion.

These results suggest that complement is critical for inducing intestinal tissue inflammation damage during IR for both sexes. All three complement initiation pathways are critical for IR-induced injury in male mice. In contrast, complement activation appears lower or delayed in female mice and is primarily initiated *via* the MBL pathway. Amplification of the complement cascade by the alternative pathway is required in both sexes. This decrease in complement activation in females is accompanied by an increase in LTB_4_ and anti-inflammatory cytokines under Sham conditions.

## Discussion

Complement activation is critical in mesenteric IR and other forms of IR-induced injury and inflammation (35, 44, 45, 56–58). Previous studies showed that intestinal, renal, and myocardial IR induced tissue damage are largely dependent on complement activation (4, 37, 50, 59, 60). Interestingly, in response to IR, the alternative complement pathway causes significant renal damage whereas intestinal and myocardial injury are more dependent on classical and MBL pathways (59). Although it is well established that IR-induced damage involves complement, the mechanistic differences between the sexes are not well understood. In humans and in other models of complement activation, complement activations appears to differ by sex (45, 61). Most IR studies used male mice as females are smaller and have less C6 and C9 activity *in vitro* (45). However, it was unknown if complement initiation in response to IR differ between sexes *in vivo*. We hypothesized that in C57B6L/mice, females may initiate complement by a different pathway than males. This study expands our previous study (24), demonstrating a sex dimorphism in the response of wildtype C57B6L/mice to intestinal IR-induced complement activation and the mechanisms were clarified in C1q^-/-^, and MBL^-/-^ mice. Our data demonstrate that although both sexes activate complement, females appear dependent on MBL initiation while males use C1q and MBL to initiate complement. While both male and female WT mice have significantly increased intestinal damage after IR, differences between sexes were apparent at multiple levels. In response to IR, intestinal transcription of *C1qa* gene and *MBL-C* genes were significantly different with significantly increased C1q in male and increased *MBL-C* in female mice, respectively (**Figures 1A, B**). During the same time points, FB gene maintained a similar transcription level in both sexes (**Figure 1C**). In contrast, at the protein level, both male and female mice increased intestinal staining of complement initiators, C1q and MBL-C in response to IR (**Figure 2**). However, deposition of both C1q and MBL were increased in males compared to females after IR while FB was similar (**Figure 2**). Thus, although previous studies agree that complement activation is critical during intestinal IR (7, 62, 63), the specific complement initiators utilized demonstrated a sex bias.

Many complement components are expressed by extrahepatic tissues including MBL-C and C1q (29, 31, 64, 65). While MBL-A, MBL-C and C1q are produced by the liver and found in the sera, intestinal epithelial and Paneth cells also produce MBL-C and C1q (29, 31, 64, 65). Thus, the current studies do not indicate the specific cell type producing the complement components. In addition, MBL-A is primarily produced by the liver as an acute phase protein, but it is only slightly elevated at eight hours post lipopolysaccharide treatment (28). Importantly, intestinal cells do not appear to produce MBL-A (29). Based on this information and our preliminary data demonstrating a lack of MBL-A, this study focused on MBL-C within the intestine. Future studies may examine the reperfusion time frame of MBL-A deposition within the intestine.

C1q recognition of antibody-antigen complexes initiates the classical pathway and MBL recognition of carbohydrate patterns triggers the MBL pathway (66, 67). A recent study shows that IgM forms complement-activating complexes with C1q on cell membranes similar to IgG1-C1 to mediate complement activation (68). Additional studies demonstrate that MBL may also recognize carbohydrate structures on the IgM (3, 69, 70). To address whether C1q and MBL play different roles in IR induced injury in different sexes, C57B6L/mice and mice genetically deficient in early components of either C1q-dependent classical or MBL pathways were characterized in intestinal model of IR damage. Male mice showed significantly lower tissue injury in absence of C1q or MBL, while females showed significantly lower damage in absence of MBL, but not C1q, at least under the conditions of our model. Previous studies show disagreement on whether mice deficient in C1q protected in distinct IR models (35, 57). The Classical pathway was robustly activated during heart and kidney ischemia (35, 57) and C1q deficiency protected animals from cerebral IR injury (71, 72). However, male C1q deficiency did not protect in 20-40 min ischemia, followed by 3 hr reperfusion in either the intestinal (3, 7) or myocardial injuries (73). In contrast, our data demonstrate that intestines from C1q^-/-^ males are protected from 30 min intestinal ischemia, followed by 2 hr reperfusion. Although female C1q-/- mice sustained some injury and statistically were not protected from IR-induced injury, when compared to wildtype female mice, all female mice were significantly protected compared to male C57B6L/mice. The difference between Hart et al. (3, 7) may be attributed to distinct reperfusion times or a different background strain. On the other hand, Zhang et al. (3, 7) did not specify animal sex but had 4 animals with significant injury and 2 without injury, suggesting a possible sexual dichotomy.

In contrast to the C1q studies, our studies with MBL^-/-^ mice are consistent with the previous studies (3, 7). Previous studies documented that MBL-deficient male mice were protected in renal (33), intestinal (3, 7), and myocardial (73) IR injuries. Similarly, we demonstrated that the absence of MBL in both male and female mice sustained significantly less tissue damage. The sexual dichotomy within complement activation is not new, as Kotimaa et al. (45) demonstrated different C6 and C9 activity in males and females despite similar C3 activation. However, our data is the first to report that the absence of MBL protects female mice. It is important to note that a lack of properdin had an intermediate effect on IR-induced intestinal injury. This may be explained by the redundancy of the complement pathways and the amplification of the alternative pathway. Together, these data suggest that male and female C57B6L/mice utilize distinct complement pathways.

The role of C5a in intestinal ischemia reperfusion injury is well established in male mice with early data suggesting that C5a is active in recruiting the inflammatory infiltrate (53, 74–76). A recent study demonstrated a role for C5a receptors 1 and 2 in activating and regulating neutrophilic infiltration in response to IR (52). Interestingly, their study demonstrated plasma C5a in male C57B6L/mice increased within 30 mins post-ischemia but was significantly decreased by 150 min post-reperfusion. However, in the current study, serum C5a increased in C57B6L/mice at 120 min post-reperfusion. These differences are likely due to differences in samples (plasma vs serum) as well as collecting the sample at room temperature and in the absence of the serine protease inhibitor nafamostat mesylate (52). These collection differences also appear to result in additional C5a activation with an increase in C5a concentrations. While these are significant differences, the male and female samples in this study were treated similarly and males produced significantly more serum C5a than females. Together, these studies suggest that while it is optimal to evaluate C5a in plasma with a serine protease inhibitor, sera C5a levels differ by sex.

The establishment of the crucial roles of complement initiation pathways in intestinal IR injury in each sex does not exclude the involvement of other innate immune component Previous studies indicated that intestinal IR resulted inTLR4 and TLR2 induction of both PGE_2_ and pro-inflammatory cytokines in male mice (22, 23). Additional recent studies demonstrate IR induces injury *via* TLR4 and subsequent signaling of MyD88 and NF/κB (55). While the authors demonstrate that dexmedetomidine inhibits IR-induced injury, they do not identify the sex of the animals (55). The current study also found increased PGE_2_, MCP-1 and TNF-α produced *ex vivo* by the male ischemic intestine and these secretions were severely reduced in the absence of any complement initiating factor. This is similar to the TLR deficient mice suggesting that TLR and complement initiation cross-talk to induce cytokine production (22, 23). Crosstalk between the two pathways was demonstrated to involve HMGB-1 and phosphoinositol-3-kinase in male mice (77). In contrast, female mice produced the neutrophil chemotactic, LTB_4_ and increased IL-10 and IL-12p40 in wildtype mice. Importantly, the lack of C1q or MBL did not significantly decrease the production of these secretions. Thus, the mechanism of TLRs in crosstalk with complement activation may differ in female mice subjected to intestinal IR. It is possible that the sexes may differ in the use of STING (stimulator of interferon genes) as a trigger of lipid peroxidation in intestinal IR. Although, the animal sex was not identified, STING appears to be critical in production of IR-induced eicosanoids, *via* Cox-2 and ALox, as well as cytokines (78, 79). These data are supported by the increase in IL-10 and IL-12p40 but not IL-12p35 in a sepsis model of LPS and C5a (80). Importantly, the TLR and complement crosstalk was not mediated by PI3K when using female mice (80). Together, these data suggest that signaling within male and female cells may differ.

In conclusion, we demonstrated that C1q-deficent male mice, but not females, were protected from intestinal IR injury at 2 hr post ischemia. In contrast MBL-deficiency protect both male and female mice in the mesenteric model. Future studies are needed to focus on MBL specific inhibitors which would protect both sexes from intestinal IR. This study also provides *in vivo* evidence that in response to intestinal IR males and females initiate distinct complement pathways and secretions leading to distinct patterns of injury. Other forms of IR (myocardial, renal or cerebral) should examine the specific differences used by each sex. Sex must be considered as an important factor in future mouse and clinical IR studies and likely for most studies.

## Data Availability Statement

The raw data supporting the conclusions of this article will be made available by the authors, without undue reservation.

## Ethics Statement

The animal study was reviewed and approved by Kansas State University Institutional Animal Care and Use Committee.

## Author Contributions

MW, JR, and SF performed the experiments, analyzed data, and wrote the manuscript and critically reviewed the manuscript. MW and SF designed experiments and directed animal experiments. SF was responsible for conception of the study and supervising the work. All authors contributed to the article and approved the submitted version.

## Funding

This work was supported by the Office of the Assistant Secretary of Defense for Health Affairs, through the Defense Medical Research and Development Program under Award No. W81XWH-18-1-0716. Additional support was obtained from National Institutes of Health P20GM103418, the H.L. Snyder Medical Research Foundation and the Johnson Cancer Research Center at Kansas State University. Opinions, interpretations, conclusions, and recommendations are those of the author and are not necessarily endorsed by the Department of Defense or National Institutes of Health.

## Conflict of Interest

The authors declare that the research was conducted in the absence of any commercial or financial relationships that could be construed as a potential conflict of interest.

## Abbreviations

IR, Ischemia reperfusion; LTB_4_, leukotriene B_4_; PGE_2_, prostaglandins E_2_; SMI, mucosal injury score; TMB, 3,3’,5,5’-tetramethylbenzidine.
