# Supplementary figures and images for: Complement Initiation Varies by Sex in Intestinal Ischemia Reperfusion Injury
Source: Front Immunol. 2021 Apr 1;12:649882. doi: 10.3389/fimmu.2021.649882 (PMC8047102; doi:10.3389/fimmu.2021.649882)

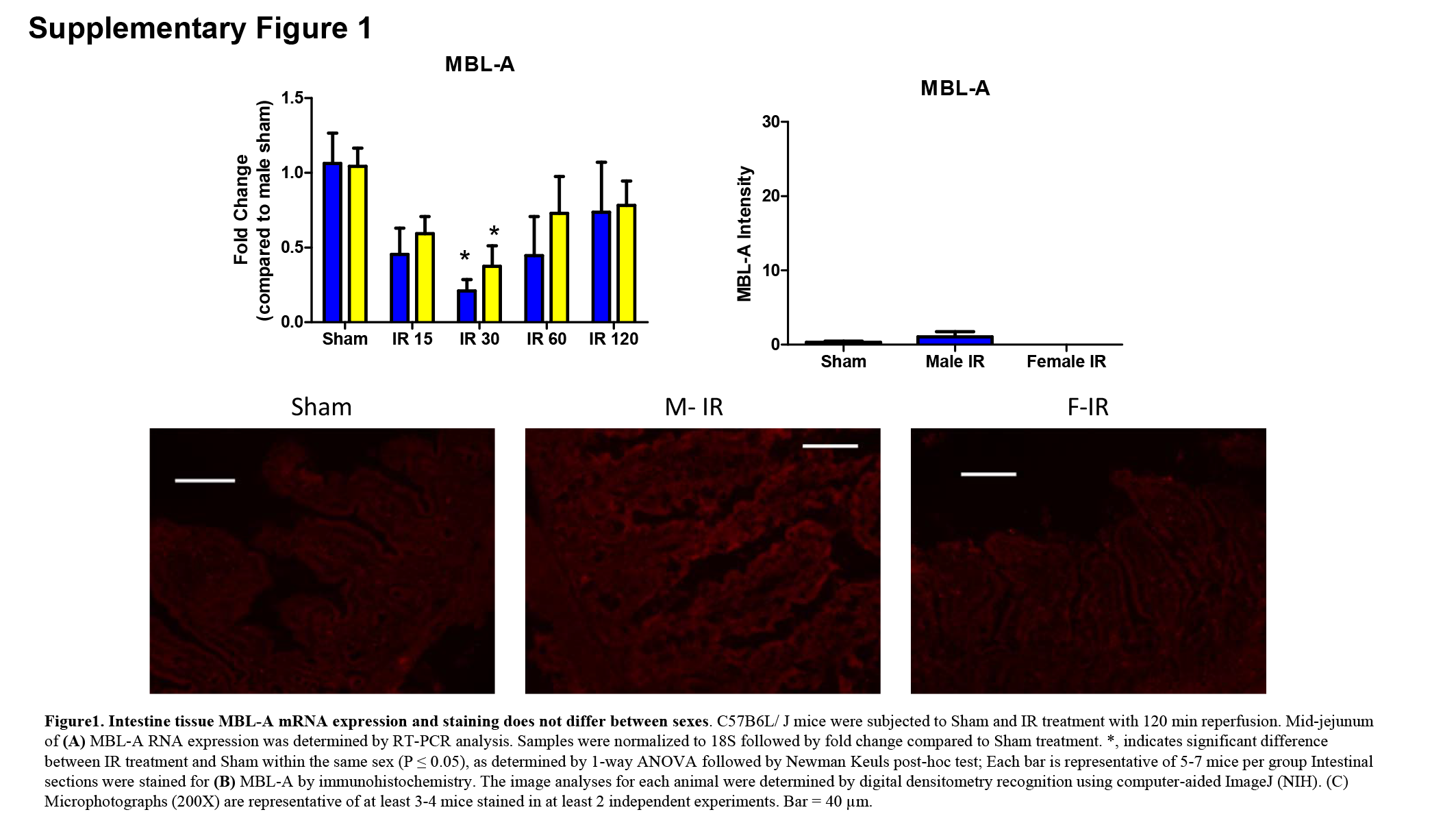

Supplement: Supplementary file 1 [file Image_1.tif]

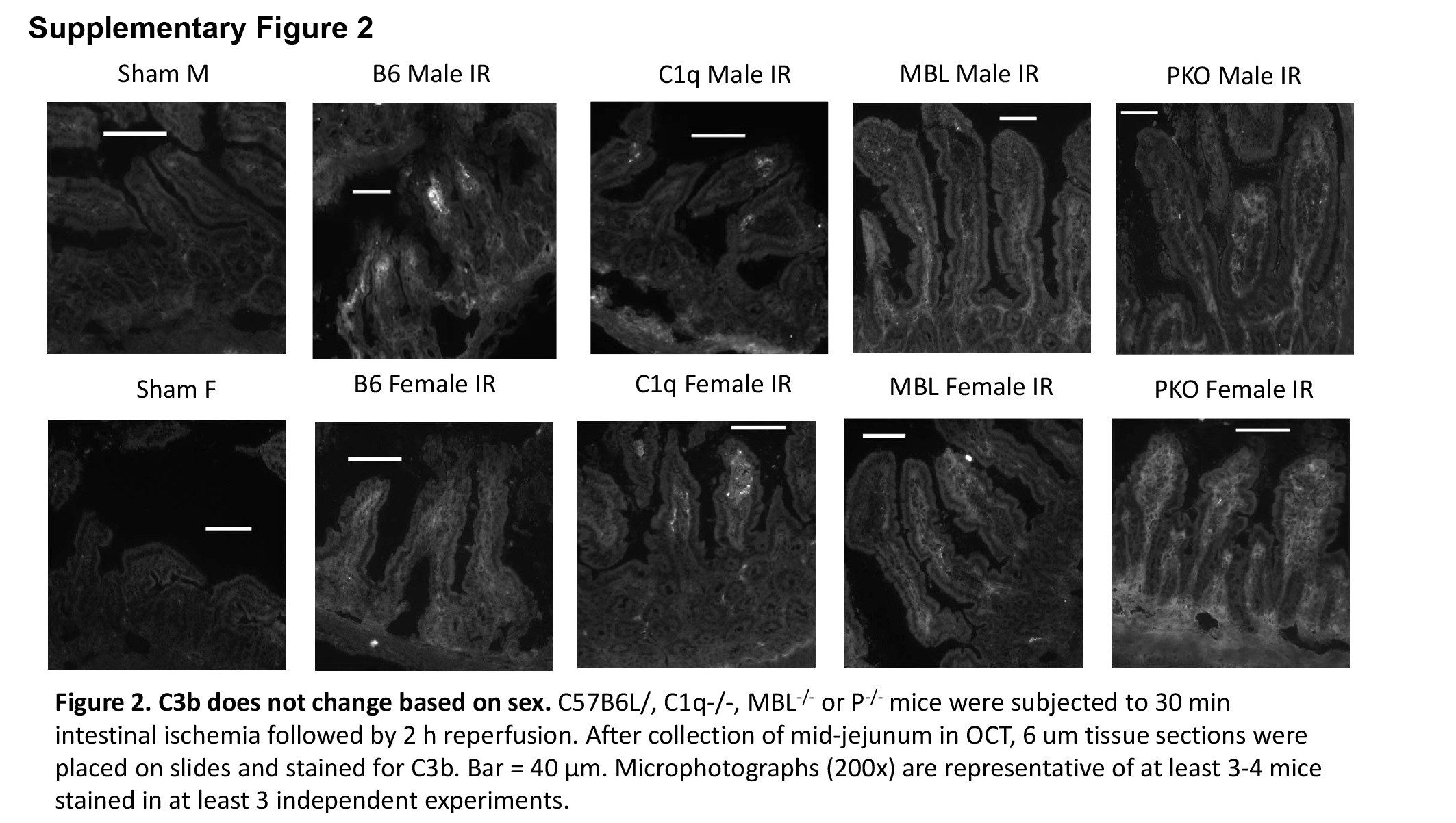

Supplement: Supplementary file 2 [file Image_2.tif]
