# Supplementary material for: Complement Initiation Varies by Sex in Intestinal Ischemia Reperfusion Injury
Source: Front Immunol. 2021 Apr 1;12:649882. doi: 10.3389/fimmu.2021.649882 (PMC8047102; doi:10.3389/fimmu.2021.649882)
Supplement: Supplementary file 3 [file Table_1.docx]

Supplementary Table 1: Sham-treated data

| Analyte | Mean ± SEM* | | | | | | | |
| --- | --- | --- | --- | --- | --- | --- | --- | --- |
|  | C57Bl/6 male | C57Bl/6 female | C1q male | C1q female | MBL male | MBL female | PKO male | PKO female |
| Injury | 0.3±0.07 | 0.03 ±0.04 | 0.5±0.04 | 0.4±0.08 | 0.6±0.1 | 0.4±0.07 | 0.4±0.03 | 0.5±0.04 |
| LTB4 | 14.8±4.8 | 8.8±1.5 | 9.2±1.9 | 7.2±1.2 | 11.84±0.6 | 6.2±1.6 | 7.6±1.5 | 6.4±2.1 |
| PGE2 | 594.7±137.3 | 644.9±62.0 | 403.6±77.8 | 637.1±246.9 | 462.3±131.1 | 270.1±17.5 | 270.0±17.5 | 504.6±177.7 |
| MCP-1 | 1.1±0.3 | 0.7±0.5 | 0.3±0.2 | 0.1±0.0 | 0.1±0.0 | 0.8±0.4 | 0.2±0.1 | 0.7±0.3 |
| IL-10 | 0.9±0.2 | 2.2±1.3 | 1.2±0.3 | 0.2±0.1 | 0.2±0.1 | 2.4±0.8 | 1.6±1.2 | 2.3±0.8 |
| IL-12p40 | 4.7±1.7 | 3.5±1.2 | 3.2±2.8 | 1.0±0.4 | 3.5± 1.2 | 3.7±3.5 | 2.7±0.9 | 1.9±1.5 |
| TNF | 0.1±0.0 | 0.4±0.1 | 0.2±0.0 | 0.2±0.0 | 0.3±0.1 | 0.3±0.0 | 0.2±0.0 | 0.1±0.0 |
| C5a | 20.3±3.0 | 20.2±4.2 | 46.00±6.2 | 25.6 ±4.2 | 30.33±5.4 | 25.4±2.9 | 15.8±1.3 | 13.63±2.8 |
| C3b | 0.6±0.6 | 1.6±0.8 | 0.9±0.5 | 0.2±0.1 | 1.7±1.4 | 1.7±1.4 | 0.3±0.3 | 1.7±1,4 |

*Standard Error of Mean

N= 3-5 animals/group
